# Toward Further Understanding of Crohn’s Disease-Related Fatigue: The Role of Depression and Emotional Processing

**DOI:** 10.3389/fpsyg.2020.00703

**Published:** 2020-04-30

**Authors:** Ingrid Banovic, Louise Montreuil, Marie Derrey-Bunel, Fabrizio Scrima, Guillaume Savoye, Laurent Beaugerie, Marie-Claire Gay

**Affiliations:** ^1^CRFDP (EA7475), Rouen Normandy University, UFR SHS, Mont-Saint-Aignan, France; ^2^CRFDP (EA7475), Rouen Normandy University, Hôpital Jacques Monod, Montivilliers, France; ^3^CHU Charles Nicolles, Rouen, France; ^4^Hôpital Saint-Antoine, Paris, France; ^5^CLIPSYD (EA4430), Paris Nanterre University, Nanterre, France

**Keywords:** fatigue, Crohn’s Disease, disease activity, emotional processing, depression, anger

## Abstract

Because the relationship between Crohn’s Disease (CD) activity and CD-related fatigue remains poorly understood, this study investigated the role of underlying psychological processes (depression, anxiety, and emotional processing). It was expected that the relationship between CD activity and CD-related fatigue would be mediated by depression and anxiety and also by a deficit in emotional processing. This prediction was tested in 110 CD patients who completed self-reported questionnaires assessing fatigue (FSS), clinical activity of Crohn’s Disease (HBAI), psychological suffering (HADS), and emotional processing (EPS-25). A path analysis showed both direct and indirect effects in the relationship between CD activity and CD-related fatigue, accounting for 33% of the variance. One indirect effect on the experience of fatigue was depression, but there was no effect of anxiety. These preliminary results confirmed that disease activity induces an increase in depressive symptoms, which in turn leads to an increase in the level of fatigue. The most novel result of the present study is that emotional processing had an indirect effect on the relationship between CD and CD-related fatigue: when the disease was more active, patients exhibited greater disruption of emotional processing, which in turn led to greater fatigue. These results did not reveal any association between depression and emotional processing. In conclusion, this work highlights the role of emotional processing in CD-related fatigue and the importance of taking this factor into account in order to manage this condition better.

## Introduction

Crohn’s Disease (CD), like all types of Inflammatory Bowel Disease (IBD), is characterized by chronic, recurrent inflammation of the gastrointestinal tract that causes the most common symptoms: diarrhea, blood and mucus in the stool, and abdominal cramping. It can also cause systemic symptoms outside the gastro-intestinal tract, such as redness or pain in the eyes, mouth sores, painful joints, skin complications, and fatigue.

Fatigue is defined as a persistent and overwhelming sense of tiredness, weakness, or exhaustion, which can be mental, physical or both (Artom et al., 2016). It is not therefore surprising that CD patients describe fatigue as one of their most troublesome symptoms (Wåhlin et al., 2019). Although its prevalence in patients with an active disease has been reported to be as high as 86%, fatigue related to CD is still poorly understood. For example, because fatigue is known to be more acute when CD is clinically active (Dignass et al., 2010; Czuber-Dochan et al., 2013b; Graff et al., 2013; van Langenberg and Gibson, 2014), it is often viewed as a direct consequence of disease activity (Opheim et al., 2014; Artom et al., 2017); and yet, CD-related fatigue actually persists in 22–41% of patients in clinical remission (Jelsness-Jørgensen et al., 2011; Czuber-Dochan et al., 2013b).

Past research addressing the issue of fatigue in CD has established a link between depression and anxiety symptoms and CD-related fatigue (Banovic et al., 2012; Mikocka-Walus et al., 2016; Neuendorf et al., 2016; Borren et al., 2019). More specifically, depressive symptoms seem to be more apparent when the disease is active or when patients present an inflammatory state (Neuendorf et al., 2016; Abautret-Daly et al., 2018). Even though depressive symptoms are – at least partly – due to the course of the disease, it is unlikely that the relationship between CD activity and fatigue can be fully mediated by these symptoms, for at least two reasons. First, the prevalence of fatigue that affects between 44 and 86% of CD patients (depending on the study and methodology) exceeds the prevalence of depressive and anxiety symptoms that are found in about 30% of these patients (Goodhand et al., 2012). Secondly, 22% of CD patients in remission still report symptoms of depression, and 41% report symptoms of anxiety (Keeton et al., 2015).

Studies of Multiple Sclerosis (MS), another inflammatory autoimmune disease, have shown that emotional processing in people with MS is more dysfunctional compared to healthy controls (Gay et al., 2019). They show how anxiety may affect depression directly, through unregulated emotions, and indirectly, by generating negative emotions (Gay et al., 2010, 2017). These results are similar to those obtained in a study with IBD patients, which found that less positive emotion recognition mediated the effects of disease activity on depression (Wilkinson et al., 2019). These difficulties in emotional processing would explain the higher rates of depression among people with these diseases. In line with Baker et al. (2010) model of emotional processing (see also Rachman, 1980), concerns associated with the daily management of CD would contribute to fatigue, taking the form of intrusive and/or repetitive thoughts that are associated with negative emotions when different stages of emotional processing (i.e., registration, appraisal, experience, awareness, and expression of emotions) are impeded. Therefore, it is plausible that the link between CD activity and fatigue is due (at least in part) to disrupted emotional processing. The outcome of CD activity would lead patients to focus on their physical condition to the detriment of taking into account their emotional states. Having relatively little direct control over the medical situation could lead to anxious ruminations and suppression of emotions in order to avoid adding emotional distress to the physical suffering. All of which would lead to a state of fatigue.

**FIGURE 1:**
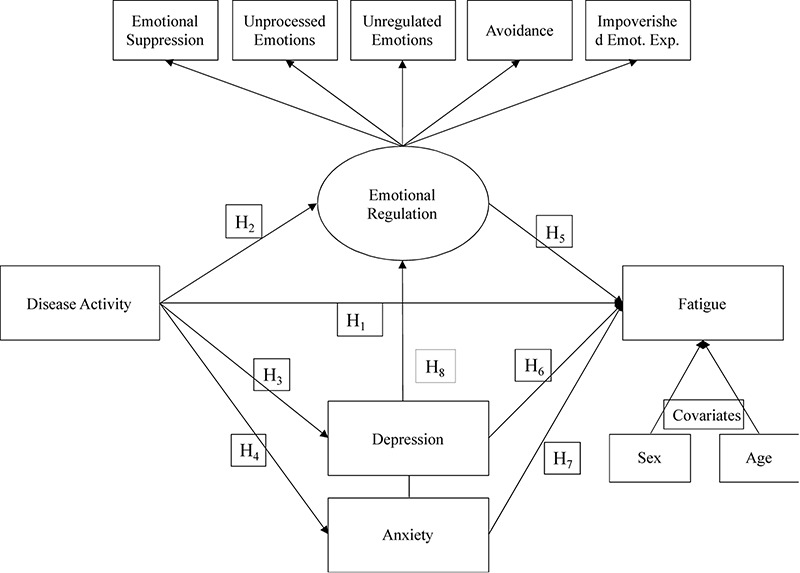
Model synthesizing the study hypotheses.

In line with past findings, e expected that the link between clinical activity of the disease and fatigue would be mediated by the severity of depression and anxiety symptoms. More importantly, we also expected to observe that emotional processing would be more dysfunctional when the disease is clinically active, leading to greater CD-related fatigue. In other words, we expected that the link between clinical activity of the disease and CD-related fatigue would be mediated by the severity of emotional dysregulation Finally, we expected these mediators to be intercorrelated and the main objective of the study was to examine their respective relative weight in the association between clinical activity of the disease and CD-related fatigue. (cf. Figure 1).

## Materials and Methods

### Participants, Recruitment and Procedure

The 110 volunteer CD patients were recruited between June 2016 and June 2017 in two hospitals: (1) a referral center for the medical management of IBD, and (2) the gastroenterology department of a teaching hospital in a medium-sized provincial city in France. They were aged 18 years or older and had a medical diagnosis of CD; 50% of the sample had an active disease (see Table 1). Participants completed the entire questionnaire when they attended the hospital for their medical treatment. The number of patients with active or inactive CD was controlled.

**TABLE 1:** Sociodemographic, health information and psychological distress (depression, anxiety, fatigue) data (number and percentage) psychological distress (depression, anxiety, fatigue) data (number and percentage).

Gender	
*Women*	61(55.45%)
*Men*	49(44.55%)
**Medical treatment**	
*Immunosuppressant*	4(3.64%)
*Anti-tnf*	67(60.91%)
*Immunosuppressant* + *Anti-tnf*	39(35.45%)
**Disease activity assessment HBAI**	
*Inactive (x* < *4)*	55(50%)
*Light (4 ≤ x ≤ 8)*	33(30%)
*Moderate (8* < *x ≤ 12)*	14(12,73%)
*Severe* (>*12)*	8(7.27%)
**Marital status**	
*Married/couple*	60(54.55%)
*Single*	38(34.55%)
*Divorced*	9(8.18%)
*Widowhood*	1(0.91%)
*Unknown*	2(1.81%)
**Education**	
*High school*	31(28.18%)
*Youth Training/BTEC First Diploma*	26(23.64%)
*College Degree or higher*	44(40%)
*No diploma*	9(8.18%)
**Employment**	
*Full-time*	58(52.73%)
*Part-time*	14(12.73%)
*Unemployed*	11(10%)
*Students*	10(9.09%)
*Without professional activity*	6(5.45%)
*Disability*	10(9.09%)
*Sick leave*	1(0.91%)
**Psychological assessment**	
- Fatigue (FSS)	
*x ≤ 4*	44(40%)
*x* > *4*	66(60%)
**- Depression (HAD-D)**	
*None (x* < *8)*	60(54.55%)
*Mild (8 ≤ x ≤ 10)*	19(17.27%)
*Moderate (11 ≤ x ≤ 14)*	28(25.45%)
*Severe (x* > *14)*	3(2.73%)
*Total of depressed patients*	50(45.45%)
**- Anxiety (HAD-A**)	
*None (x* < *8)*	34(30.91%)
*Mild (8 ≤ x ≤ 10)*	23(20.91%)
*Moderate (11 ≤ x ≤ 14)*	36(32.73%)
*Severe (x* > *14)*	17(15.45%)
*Total of anxious patients*	76(69.09%)

In accordance with French legislation and the Declaration of Helsinki, participants were fully informed about the purpose of the study, which was approved by the Ile-de-France Ethical Research Committee (no. 2015-09-05). Once all their questions about the study had been answered and they had given their written consent, they completed (in 25 min) a paper-and-pencil questionnaire containing the measures outlined below.

### Measures

*Demographic and disease-related information* included age, gender, marital status, years of education, employment status, and medical treatment.

*Clinical activity of* CD was assessed with the modified Harvey-Bradshaw activity index (HBAI; Harvey and Bradshaw, 1980) for CD patients. Patients with HBAI scores ≤ 4 were classified as having an inactive disease.

*Fatigue* was assessed with the 9-item Fatigue Severity Scale (FSS). Items are rated on a 7-point scale ranging from (1) “disagree” to (7) “agree”; the fatigue threshold score is set at 36. The mean of the scores for the nine items represents a continuous variable with values ranging from 1 (no fatigue interference) to 7 (maximum fatigue interference). This scale has good psychometric properties (Whitehead, 2009) and is therefore one of the most commonly used self-report questionnaires to measure fatigue (Krupp et al., 1989; Opheim et al., 2014).

*Anxiety and depression* were assessed with the Hospital Anxiety and Depression Scale (HADS), which is a 14-item scale used as a brief instrument for detecting the intensity of depression and anxiety in patient populations (Zigmond and Snaith, 1983; Herman, 1997). The HADS has few somatic items so is unlikely to confuse depression with physical symptoms such as pain and fatigue. Scores for the depression and anxiety subscales can range from 0 to 21, with a score >10 indicating probable anxiety or depression. The French adaptation of the HADS confirmed Zigmond and Snaith’s original two-factor structure and has been shown to possess good psychometric properties. Internal reliability ranges from 0.79 to 0.90 for the anxiety subscale, and from 0.79 and 0.90 for the depression subscale. The correlation between the two subscales is significant but moderate (*r* = 0.46), representing 22% of the common variance (Lépine et al., 1985).

*Emotional processing* was assessed with the Emotional Processing Scale (EPS-25), which is a 25-item self-report questionnaire designed to identify and measure emotional processing styles and potential deficits in healthy individuals and those with psychological or physical disorders. It comprises five subscales, each with five items that are rated on a 10-point (0–9) attitudinal scale: suppression (excessive control of emotional experience and expression), signs of unprocessed emotion (intrusive and persistent emotional experiences), unregulated emotion (inability to control one’s emotions), avoidance (avoidance of negative emotional triggers), impoverished emotional experience (detached experience of emotions due to poor emotional insight). Total score can range from 0 to 225, higher scores indicating poorer emotional processing. In the original English version of the EPS developed in the United Kingdom, these five factors explained 59.4% of the total variance, and overall internal reliability was high (α = 0.92), ranging from 0.70 to 0.80 for the five factors. A French version has been developed (Gay et al., 2019).

### Data Analysis Plan

First, some preliminary analyses were conducted (Student’s *t*-test, correlations). To gain a better understanding of the characteristics of the sample, the differences in the mean scores of participants with active and inactive disease were calculated using Student’s *t*-test. Next, to explore the bivariate relationships between the variables under study, zero-order correlations were calculated. Our research hypotheses were tested through a multiple mediation model using Amos 22 software. We evaluated model fit using the following indices: Carmines-McIver Index (χ^2^/df) (Carmines and McIver, 1981), the Non-Normed Fit Index (NNFI) (Jöreskog and Sörbom, 1996), the Comparative Fit Index (CFI) (Bentler, 1990), and the Root Mean Square Error of Approximation (RMSEA) (Browne and Cudeck, 1993). If the Carmines-McIver Index, which is the ratio between χ2 and degrees of freedom, has a value less than or equal to three, this indicates an excellent fit. For the other indices, to assess the adequacy of a model, Hu and Bentler (1999) proposed threshold values greater than or equal to 0.90 for the NNFI and CFI, and values less than or equal to 0.08 for the RMSEA. Finally, to verify the mediation effect, total and specific indirect effects were estimated using the bootstrap bias-correction method.

## Results

### Patients

Participants had a mean age of 38.04 years (*SD* = 12.06), with a slight predominance of women (55.45%); the majority were married or living with a partner (54.55%), and 65.46% had a professional activity. 50% had a clinically active disease, and for 60% of those, the clinical activity was light.

In our sample, 60% reported a moderate fatigue (*M* = 4.55, *SD* = 1.69), and 69.09% reported anxiety. While patients reported moderate levels of anxiety, the prevalence in the sample was 69.09%. Overall level of anxiety was low (*M* = 9.94, *SD* = 4.68). Mean depression scores were low (*M* = 6.89, *SD* = 4.45), although 45.45% of patients reported symptoms of depression.

Regarding emotional processing (see Table 2), the EPS-25 total score indicated dysfunctional processing (*M* = 3.65, *SD* = 1.88) compared to the general population (*M* = 2.51, *SD* = 1.04), with the mean of the clinical sample nearing two standard deviations below the mean of healthy controls. All subscale scores were significantly different from those obtained by healthy controls (Gay et al., 2019), whether the disease was active or not*:* suppression (*M* = 4.89, *SD* = 2.37 vs. *M* = 3.38, *SD* = 2.35), avoidance (*M* = 4.26, *SD* = 2.10 vs. *M* = 3.29, *SD* = 1.97), unprocessed emotions (*M* = 4.92, *SD* = 2.39 vs. *M* = 3.85, *SD* = 2.47), unregulated emotions (*M* = 3.94, *SD* = 2.06 vs. *M* = 2.74, *SD* = 2.35), and impoverishment (*M* = 3.34, *SD* = 2.14 vs. *M* = 2, *SD* = 1.89). The average scores obtained on the unprocessed emotions dimension were particularly high, indicating intrusive and persistent emotional experiences, which may be an indicator of a traumatic experience. Suppression of emotions also reflects the cost of trying to deal with emotions for these patients.

**TABLE 2:** Psychological assessments (mean scores and SD) for the whole sample and significant differences between patients with active and inactive disease.

	All sample mean (SD)	Active disease mean (SD)	Inactive disease mean (SD)	*t*	*p*
Age	38.04(12.06)	38.84(12.64)	37.25(11.53)	0.68	n.s.
HADS-D	6.89(4.45)	8.49(4.01)	5.29(4.32)	4.02	<0.001
HADS-A	9.94(4.68)	10.51(4.14)	9.36(5.14)	1.28	n.s.
FSS	4.55(1.69)	5.21(1.36)	3.88(1.68)	4.47	<0.001
Emotional processing	3.65(1.88)	4.27(1.73)	3.05(1.84)	3.50	<0.001
*- Suppression*	4.12(2.47)	4.89(2.37)	3.38(2.35)	3.30	<0.001
*- Unprocessed emotion*	4.38(2.44)	4.92(2.39)	3.85(2.47)	2.29	<0.05
*- Unregulated emotion*	3.33(2.29)	3.94(2.06)	2.74(2.35)	2.77	<0.01
*- Avoidance*	3.76(2.08)	4.26(2.10)	3.29(1.97)	2.45	<0.01
*- Impoverished emotion*	2.66(2.12)	3.34(2.14)	2(1.89)	3.42	<0.001

### Comparison of Patients With a Clinically Active and a Clinically Inactive Disease

Results showed that there was no significant difference between anxiety scores of patients with an active disease and those with no disease activity: both groups reported clinical-level anxiety, with no decrease when the disease was no longer active. There were significant differences in fatigue and depression in relation to disease activity. Patients with an active disease reported significantly more fatigue and depression (see Table 2).

Regarding emotional processing, patients with an active disease had significantly more difficulty on all dimensions of emotional processing. Compared to patients with a clinically inactive disease, they showed greater avoidance of emotional triggers, had a more detached experience of emotions, exerted more control of emotional experience and expression, and showed a higher tendency to express anger.

Finally, it is important to note that scores for avoidance, emotional suppression and unprocessed emotion were particularly high in the active disease group. In other words, these patients had impoverished emotions, avoided negative stimuli, perceived events as significantly more external to them, and experienced more anger than patients with an inactive disease.

### Bivariate Relations Between Variables

We analyzed the bivariate relationships between age, sex, Disease Activity, depression, anxiety, the total score of the emotional processing variable and its five dimensions (Emotional suppression, Unprocessed emotions, Unregulated emotions, Avoidance, Impoverished emotional experience) and fatigue. The results are shown in Table 3. Gender was positively associated with the total fatigue score (*r* = 0.25, *p* < 0.01), women having higher scores than men. The younger participants had higher scores for anxiety (*r* = −0.19, *p* < 0.05) and emotional suppression (*r* = −0.19, *p* < 0.05). Furthermore, Disease activity was positively correlated with depression (*r* = 0.36, *p* < 0.01), emotional processing (*r* = 0.32, *p* < 0.01), and fatigue (*r* = 0.39, *p* < 0.01), but not with anxiety (*r* = 0.12, *p* = n.s.). In fact, the only variable that correlated significantly with anxiety was fatigue. Finally, emotional processing was positively correlated with fatigue (*r* = 0.46, *p* < 0.01).

**TABLE 3:** Pearson’s *r* correlations of variables (*n* = 110).

		1	2	3	4	5	6	7	8	9	10	11	12
1	Sex (0 = F, 1 = M)	1											
2	Age	0.006	1										
3	Disease activity (0 = Not, 1 = Yes)	–0.018	0.066	1									
4	HADS-D	–0.088	–0.186	0.361**	1								
5	HADS-A	0.153	−0.194*	0.123	0.517**	1							
6	Emotional processing (Total)	0.091	0.003	0.325**	0.238*	0.160	1						
7	*– Emotional suppression*	0.011	−0.188*	0.308**	0.302**	0.056	0.790**	1					
8	*– Unprocessed emotions*	0.166	0.076	0.219*	0.143	0.150	0.858**	0.636**	1				
9	*– Unregulated emotions*	0.024	–0.026	0.263**	0.214*	0.141	0.793**	0.475**	0.627**	1			
10	*– Avoidance*	0.187	0.103	0.234*	0.129	0.112	0.831**	0.525**	0.628**	0.559**	1		
11	*– Impoverished emotional experience*	–0.011	0.072	0.318**	0.184	0.149	0.865**	0.589**	0.628**	0.625**	0.775**	1	
12	FSS_(Total)	0.249**	0.033	0.395**	0.414**	0.257**	0.465**	0.374**	0.415**	0.319**	0.445**	0.370**	1

*p < 0.05; **p < 0.01.

### Testing the Multiple Mediation Model

**FIGURE 2:**
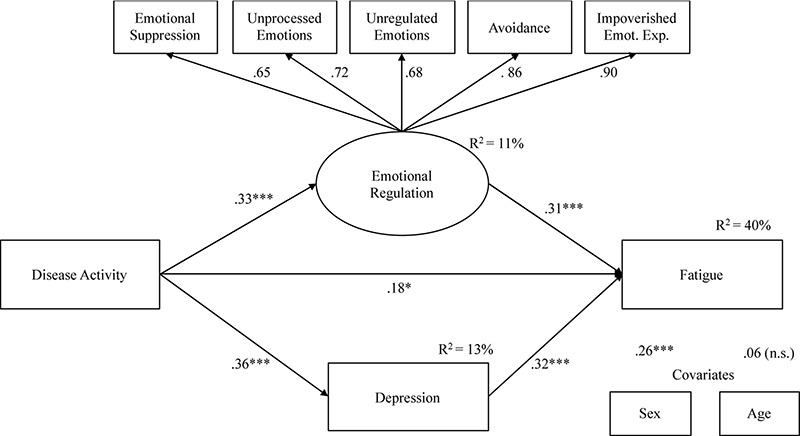
Mediation model: indirect effects of Disease Activity on Fatigue through Emotional processing and Depression.

Figure 2 shows our final model, without the anxiety variable. Although an anxiety-mediating effect was hypothesized in the relationship between Disease activity and Fatigue, we were unable to obtain a model that showed adequate fit indices and with significant structural parameters of the relationship between Disease activity and anxiety and between anxiety and fatigue. Therefore, as partially expected, our results showed a partial mediation model, indicating suitable fit indexes: X^2^ = 24.16, df = 22, X^2^/df = 1.10, NNFI = 0.98, CFI = 0.99, and RMSEA = 0.03 (Low = 0.00, High = 0.089). Disease activity was positively associated with fatigue (β = 0.18, *p* < 0.01), depression (β = 0.36, *p* < 0.001), and Emotional regulation (β = 0.33, *p* < 0.001). Moreover, emotional regulation (β = 0.31, *p* < 0.001) and Depression (β = 0.32, *p* < 0.001) were associated with fatigue. The model explained 40% of fatigue variance.

Table 4 shows total and specific indirect effects of the model. The 95% bootstrap confidence interval for the total indirect effect did not include zero (C.I. = 0.064;0.338), as well as confidence intervals for specific indirect effects (C.I. = 0.032;0.204 for Emotional regulation, and C.I. = 0.057;0.217 for Depression), suggesting a significant partial mediation effect.

**TABLE 4:** Total and specific indirect effect of mediation model.

Total indirect effect	Product of coefficients			Bootstrapping bias-corrected 95% CI	
Relationship	Estimate	SE	*p*	Lower limit	Upper limit
Disease activity to fatigue	0.220	0.064	<0.001	0.125	0.338
Emotional regulation	0.116	0.047	<0.001	0.057	0.217
Depression	0.103	0.052	<0.001	0.033	0.204

## Discussion

The main objective of this research was to examine the role of specific psychological factors (anxiety, depression, and emotional processing) underlying CD-related fatigue.

These preliminary results confirmed all the predictions, except the expected positive link between disease activity and anxiety. This could be explained by the prevalence of CD patients with anxiety symptoms in our sample (69.09%, which is high but still within the upper limits observed elsewhere: Mikocka-Walus et al., 2016; Neuendorf et al., 2016). This suggests that CD patients experiencing anxiety are caught in a vicious circle, in which disease activity does not worsen but only confirms their negative expectations. The other aspects of the psychological profile of CD patients were consistent with the literature: half of the patients had depressive disorders [which is high, but, like anxiety, is within the levels observed elsewhere, cf. Mikocka-Walus et al., 2016; Neuendorf et al., 2016], and two-thirds showed pathological anxiety. The EPS-25 total score indicated dysfunctional processing compared to the general population, with the mean of the clinical sample nearing two standard deviations below that of healthy controls. CD patients had great difficulty dealing with their emotions, with extensive emotional avoidance, suppression and impoverishment. Their scores on the subscales of unregulated emotion (whose main manifestation is anger) and unprocessed emotions (whose manifestations are rumination and intrusive thoughts) also suggest poor emotional processing. When the disease was active, patients reported significantly more fatigue and depression; their difficulty dealing with emotions increased significantly, suggesting a traumatic state with greater reactivity to and dysfunctional control of emotional stimuli.

The present preliminary study thus indicates that disease activity, depression, and emotional processing all predict CD-related fatigue. Moreover, and importantly, the study found both direct and indirect relations between disease activity and fatigue. First, in line with past studies (Czuber-Dochan et al., 2013a; Huppertz-Hauss et al., 2017), the relationship between CD activity and CD-related fatigue was mediated by the severity of depressive symptoms. Secondly, the most novel finding of the present study is that the relationship was also mediated by the efficiency of emotional processing. This two-way model, which explained 40% of the variance, could be consistent with the idea of the overwhelming consequences of disease activity, which simultaneously is related to psychological breakdown (i.e., depression) or to impaired emotional processing in many individuals could explain a part of CD related fatigue. Lastly, the most surprising result is the lack of link between depression and emotional processing in the multiple mediation model tested. From a theoretical point of view, this lack is unexpected because depression is related to dysfunctional emotional regulation processing (Rachman, 1980; Teasdale, 1999; Trindade et al., 2017).

The present preliminary results shed further light on the observation that some CD patients continue to experience fatigue when they are in remission. Our results confirm that this fatigue is likely to be maintained not only by the persistence of depressive symptoms, but also, and importantly, by poor emotional processing of the negative experiences that are inevitably associated with medical conditions like CD. Indeed, while remission might allow some patients to regulate their emotions effectively and thus to implement effective psychological adjustment strategies, for others, somewhat ironically, remission may increase anxiety about possible relapse. This might explain the ongoing difficulty dealing with their emotions and why CD-related fatigue persists or even worsens. However, future research is required to address these possibilities, both in CD and in other chronic medical conditions (Menting et al., 2018).

The present study has a number of limitations. The lack of a control group and of a second assessment prevents a detailed understanding of how and to what extent emotional processing is impaired by the clinical activity of the disease, and how and to what extent experiencing a chronic disability disease disrupts emotional processing. It thus remains possible that reverse effects could be observed in patients with a disease that becomes inactive, and/or in patients who are less impaired by the severity of CD-related fatigue. A longitudinal study should therefore be conducted to better understand how patients deal with fatigue and emotional processing over time, which may also provide a better understanding of the role of anxiety over time. Finally, emotional processing and depression in CD patients could be mediated by other relevant psychological factors such as coping strategies. Emotional processing would probably play as essential a role in depression as the modalities of patients’ psychological adjustment. Patients would have to deal with physical difficulties and self-related negative cognitions due to the discrepancies between their actual and desired states (Czuber-Dochan et al., 2013b; Wåhlin et al., 2019). Depression could occur not only because of dysfunctional processing of emotions (Wilkinson et al., 2019), but also because of inoperative coping strategies leading to emotional overload, which would lead to increased fatigue. Further research is necessary to test a causal and more complex model of CD related fatigue. Despite these limitations, the present study clearly points to emotional processing as an important factor in the relationship between CD activity and CD-related fatigue. It would also be important to consider the duration of the disease (taking account relapses, major changes in the management of the CD for example) and the time of the disease’s onset (childhood, adolescence, adulthood), as these may have outcomes on emotional processing and on fatigue (Hughes et al., 2012; Mikocka-Walus et al., 2016). A better understanding of how CD patients process their emotions could also clarify why some patients appear more resilient to CD-related depression and fatigue than others.

Given this newly established role of emotional processing, it is likely that acceptance and commitment therapy could offer effective management strategies for CD patients (Wynne et al., 2019). This would help individuals to experience their emotions, thoughts, and bodily sensations fully without trying to change, control, or avoid them (Hayes et al., 1999). Several recent studies have shown that this approach can lead to positive outcomes in individuals with depression and anxiety (Twohig and Levin, 2017). Acceptance can also help individuals with chronic pain and fatigue to adjust and function better (Hughes et al., 2017; Jacobsen et al., 2017). It would therefore be very relevant for future research to investigate the extent to which acceptance and commitment therapy could help CD patients cope with depression and fatigue.

## Data Availability Statement

All datasets generated for this study are included in the article/supplementary material.

## Ethics Statement

The studies involving human participants were reviewed and approved by the Ile-de-France Ethical Research Committee (no. 2015-09-05). The patients/participants provided their written informed consent to participate in this study.

## Author Contributions

IB and MC-G: substantial contributions to the conception or design of the work, analysis or interpretation of data for the work. LM and MD-B: the acquisition of data for the work and they organized the database. FS: analysis or interpretation of data for the work, he performed the statistical analysis. LB and GS: substantial contributions to the acquisition of data for the work. IB, FS, MC-G, GS, and LB wrote sections of the manuscript. All authors contributed to manuscript revision, read and approved the submitted version.

## Conflict of Interest

The authors declare that the research was conducted in the absence of any commercial or financial relationships that could be construed as a potential conflict of interest.
